# The mapping of eccentricity and meridional angle onto orthogonal axes in the primary visual cortex: an activity-dependent developmental model

**DOI:** 10.3389/fncom.2015.00003

**Published:** 2015-01-29

**Authors:** Ryan T. Philips, V. Srinivasa Chakravarthy

**Affiliations:** Computational Neuroscience Laboratory, Department of Biotechnology, Indian Institute of Technology MadrasChennai, India

**Keywords:** development, complex-logarithmic, retinotopy, self-organizing, LISSOM, V1, plasticity

## Abstract

Primate vision research has shown that in the retinotopic map of the primary visual cortex, eccentricity and meridional angle are mapped onto two orthogonal axes: whereas the eccentricity is mapped onto the nasotemporal axis, the meridional angle is mapped onto the dorsoventral axis. Theoretically such a map has been approximated by a complex log map. Neural models with correlational learning have explained the development of other visual maps like orientation maps and ocular-dominance maps. In this paper it is demonstrated that activity based mechanisms can drive a self-organizing map (SOM) into such a configuration that dilations and rotations of a particular image (in this case a rectangular bar) are mapped onto orthogonal axes. We further demonstrate using the Laterally Interconnected Synergetically Self Organizing Map (LISSOM) model, with an appropriate boundary and realistic initial conditions, that a retinotopic map which maps eccentricity and meridional angle to the horizontal and vertical axes respectively can be developed. This developed map bears a strong resemblance to the complex log map. We also simulated lesion studies which indicate that the lateral excitatory connections play a crucial role in development of the retinotopic map.

## 1. Introduction

A mapping scheme from an abstract visual input space to the cortical space, which is topology-preserving to a certain extent is an organizational feature of the striate cortex (V1) in primates. In fact, a number of such mapping schemes, which overlap to varying degrees, are observed in V1 (Schiller et al., 1976; Hubel and Wiesel, 1977; Tootell et al., 1988). It is speculated that the retinotopic, orientation, ocular dominance, motion direction maps are overlaid in such a manner that they resemble a uniform distribution across these features (Swindale et al., 2000). The hypercolumn, which is the basic functional unit of the V1 architecture, is composed of cortical columns that are orientation selective, and these orientation columns span the range of all possible orientations. Each hypercolumn has a dimension of approximately 0.4 mm^2^ (Hubel and Wiesel, 1977), and these hypercolumns span the surface area of each hemisphere (approximately 1380 mm^2^ for the rhesus monkey) of the striate cortex. Thus, from a global perspective, each of these hypercolumns can be approximated to a point in the cortical space. With this approximation in place, a mapping function from a point (*x*, *y*) in the visual field to a point (*u*,*v*) in the cortical space has been proposed (Schwartz, 1977):

(1) $$x+iy=re^{i\theta }$$

**Figure 1:**
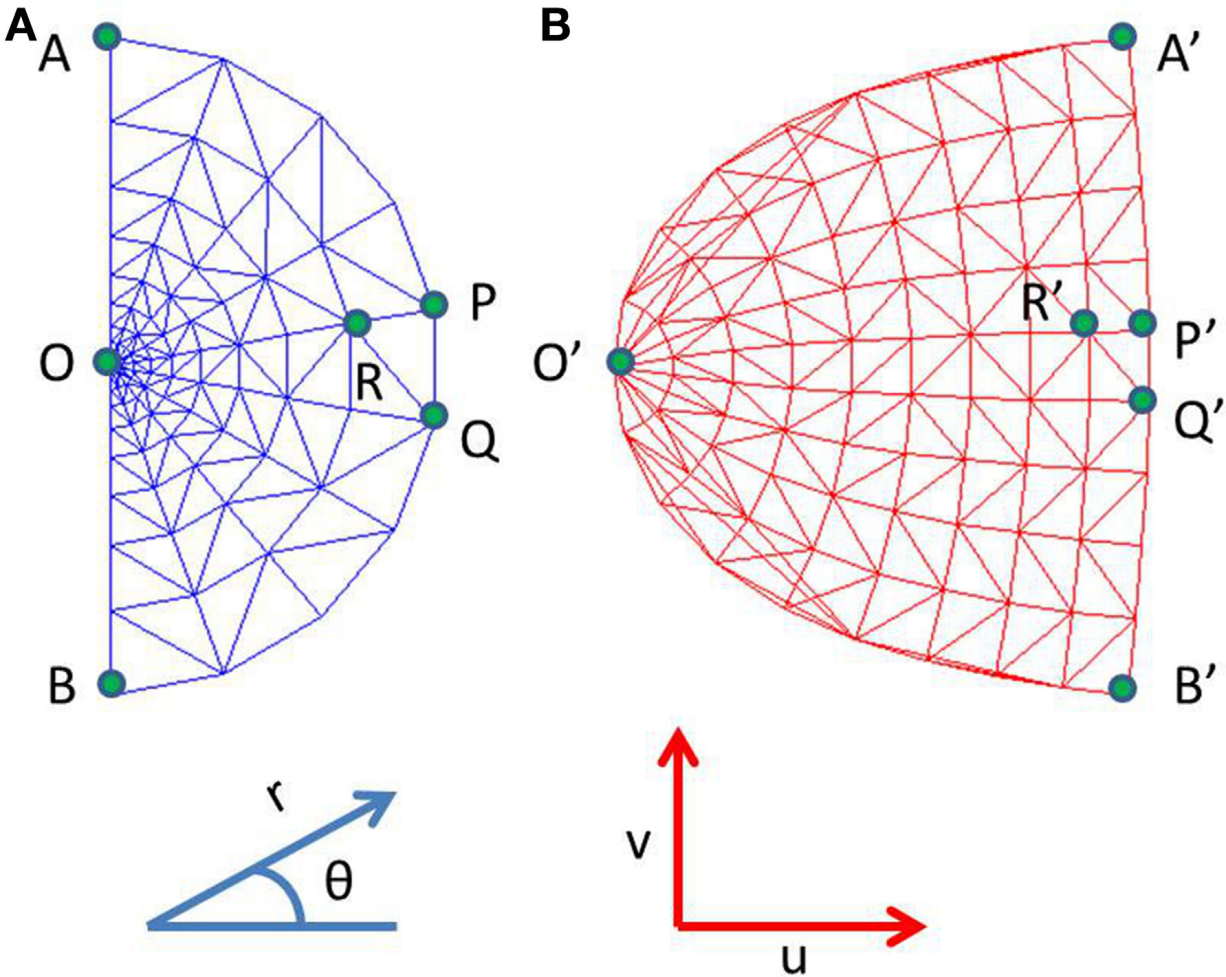
**Complex logarithmic transformation. (A)** Each vertex represents a certain location in the visual field; **(B)** Each vertex represents the corresponding transformed location in the cortical space.

(2) u+iv=log(x+iy+a)

where *x*, *y* are Cartesian co-ordinates for a point in the visual field, and *r* and θ are the corresponding Polar co-ordinates representing eccentricity and meridional angle respectively; *u* and *v* are the Cartesian co-ordinates of a point in the cortical space; *a* is a constant. By varying the value of *a*, the retinotopic mappings in a number of primate species have been approximated (Schwartz, 1980). This transformation is illustrated in Figure 1 for the central 4° of eccentricity, with the value of *a* chosen to be 1. The retinotopic map is one of the first maps approximated by a mapping function. It describes the relationship between a point in the visual field and its representation in the cortical space. While the logarithmic nature of the map along the nasotemporal (*u*) axis is probably a consequence of the exponential decay in the density of Retinal Ganglion Cells (RGCs) from the fovea radially outwards (Wässle et al., 1990), it does not explain the map formation along the dorsoventral (*v*) axis. Schwartz et al. (Schwartz, 1977) speculated that this kind of mapping would result in the rotational and dilational variance in the input space to be transformed to a translational variance along both axes in the output space.

The development of such a retinotopic mapping is of current experimental interest. A fairly comprehensive retinotopic map is believed to be present even prior to eye opening, that is prior to inputs arriving from rod and cone photo-receptors (Espinosa and Stryker, 2012). There are two major mechanisms, known primarily from the mouse model, which contribute to this map formation, namely molecular gradients and retinal waves. Post cortical arealization, wherein neurons in the striate cortex establish their identity, axon terminals from LGNd project to the V1. The EphA family of receptors are expressed on the axon terminals of these LGNd axons. Now a molecular gradient of the ephrin-A ligand, which is attached to the surface of neurons, is present along the nasotemporal axis of the V1. This EphA ephrin-A interaction is one of the mechanisms for the development of the retinotopic map along the nasotemporal axis (Cang et al., 2005). Retinal waves, caused by the spontaneous firing of retinal ganglion cells (RGCs), constitute another factor that influences the development of the retinotopic map along the nasotemporal axis (Wong et al., 1993). On disrupting both ephrin-As and retinal waves, the map along the nasotemporal axis is almost completely eliminated, however the map along the dorsoventral axis of V1 is more or less maintained (Cang et al., 2008). This has led to the speculation, that there are two distinct mechanisms for the development of the retinotopic map along the dorsoventral and nasotemporal axes.

There is also evidence of plasticity in the retinotopic map, both during development as well as in adulthood. This plasticity could be classified into two categories: plasticity in the afferent connections from the retina to V1, plasticity in the lateral connections withing V1 itself. Visual experience driven map refinement shortly after eye opening falls in the first category (Smith and Trachtenberg, 2007). There is debate whether the plasticity seen in rod monochromats falls in the first category or the second (Wandell and Smirnakis, 2009). Rod monochromats, who have a defect in cone transduction, exhibit fmri activity in the central 1 cm^2^ region of the V1, in response to rod driven signals (Baseler et al., 2002). In normal subjects however this entire region in unresponsive for the same stimuli. Thus, there is a rod projection zone seen in a cortical location which in normal subjects corresponds to a cone-only projection zone. This kind of plasticity is photo-receptor driven and hence most probably occurs post eye-opening.

Another kind of plasticity is seen in adults with binocular retinal lesions. The radius of the observed plasticity is reduced. Different groups performing lesion studies on cats and monkeys report activity up to 8 mm within the lesion projection zone (LPZ) (Kaas et al., 1990; Schmid et al., 1996; Eysel et al., 1999). This radius corresponds to the known radius of lateral connections within V1, which has led to speculation that adult cortical plasticity is limited by the radius of horizontal lateral connections in V1. This kind of plasticity probably falls in the second category.

Various computational models have been proposed to explain the development of other visual maps in V1, like orientation maps and ocular-dominance maps. One family of models known as the General Elastic Nets (GENs) (Durbin and Willshaw, 1987), is based on an optimization criterion, such that the resulting map is optimized for the continuity of the output representation of input features, as well as the coverage of output space corresponding to each input feature. In another family of models known as Self Organizing Maps (SOMs) (Willshaw and Von Der Malsburg, 1976), the map development is based on a local correlational learning mechanism. Previously proposed self organizing models do not capture this unique mapping of eccentricity and meridional angle onto orthogonal axes. They merely ensure a topography preserving map in the output cortical space (Kohonen, 1982). Some SOM models simulate the effect of a boundary condition on the final nature of the map developed (Wolf et al., 1994), though the map developed is not very accurate when compared with the theoretically estimated complex log map. A more biologically realistic variant of the SOM architecture has been proposed namely the Laterally Interconnected Synergetically Self Organizing Map (LISSOM) (Sirosh and Miikkulainen, 1994; Miikkulainen et al., 2005) which is the framework employed for the development of the retinotopic map, described in this paper. A more complete description of both the SOM and LISSOM architectures is given in the methods section.

The neurons in V1 are known to receive inputs from a number of locations, such as the lateral geniculate nucleus (LGN), extrastriate cortex, middle temporal (MT) region, other V1 neurons, etc. In order to model the development of the retinotopic map, in this paper only three kinds of connections are considered: connections from the RGCs to the V1 (via the LGNd), lateral excitatory connections within V1, and lateral inhibitory connections within V1. The LISSOM architecture proves ideal for this kind of simulation, since all these three kinds of connections are available in the LISSOM framework. The strengths (weights) of these connections are updated in LISSOM using a normalized Hebbian mechanism. In this paper, an activity based model for the development of the retinotopic map is described, using the aforementioned SOM and LISSOM architectures. It is proposed that a training regime with rotated and dilated rectangular bars as inputs, could drive the refinement of the retinotopic map, modeled using a LISSOM based architecture.

Although molecular mechanisms are also involved in the formation of the retinotopic map, the model described in this paper focuses exclusively on the activity-dependent mechanisms. Only the initial rough topography imposed on the LISSOM architecture, is assumed to be present as a result of a chemical gradient mechanism. In the LISSOM model, this rough topography by itself does not result in the mapping of eccentricity and meridional angle onto orthogonal axes (See Supplementary Material Figure 3).

The initial SOM simulations were performed primarily to demonstrate that the two abstract features of an object in an image, namely its dilation and rotation would be mapped onto distinct axes by a self-organizing mechanism. Thus, the SOM model is presented as a preliminary model, which paves the way for the LISSOM model. In order to develop a more accurate output map comparable with the theoretical estimate, utilizing a more biologically realistic architecture, the LISSOM based simulations are performed.

## 2. Methods

As mentioned earlier, the complex log map proposed by Schwartz (1977), maps eccentricity and meridional angle to orthogonal axes closely resembling experimental observations. Why did the visual system map eccentricity (scale) and meridional angle (rotation) onto orthogonal axes? Is it possible to explain this phenomenon as a result of activity-dependent plasticity driven by visual stimuli? The key insight underlying the proposed model is as follows: We assume that, due to relative motion, objects are seen by the visual system at various angles (due to relative rotation around the line of sight) and at various scales (due to relative translation along the line of sight). Thus, it is possible that when the same object is presented repeatedly at different scales and angles, the visual system might map, by some process of self-organization, the two properties along two different axes. In order to test this notion, we present rectangular bar patterns at various scales and angles to a self-organizing learning system and observe how scale and rotation are mapped onto the output space. The presentation of the rectangular bars to the model is done in a random order, i.e., at any iteration any rectangular bar of any dilation or rotation could be presented to the model as an input. Thus, training of the system is performed using rotated and scaled versions of whole patterns. However, since the retinotopic map is a point-to-point map between the visual and cortical spaces, ‘point’ inputs are presented to the trained system, and ‘point’ responses are observed. The map thus generated is found to represent eccentricity and meridional angles onto orthogonal axes. In this section the architecture of the SOM and the LISSOM models, used in simulating the development of the retinotopic map are described in brief.

Any self-organizing architecture maps similar inputs to near by (adjacent) locations in the output space. In the case of the rectangular bars, there are two features namely their dilation and rotation which are considered in order to establish similarity between the inputs. Now since these two features are completely independent of each other, a self-organizing mechanism would map them onto roughly orthogonal axes, in order to maximally utilize the output space.

The Willshaw-von der Malsburg SOM model (Willshaw and Von Der Malsburg, 1976) was initially proposed to demonstrate that correlated activity in the input (retinal) layer, could result in a topography preserving map in the output (tectal) layer. A simplified (in terms of architecture) and more generalized (in terms of input dimensions) version of SOM was proposed by Kohonen (1990). The basic framework for a 2 dimensional output version of Kohonen's SOM is described below.

The SOM model consists of 2 layers: an input layer and an output layer. There are connections between every neuron in the input layer with every neuron in the output layer. The output layer for a 2D-SOM is arranged as a regular rectangular grid of neurons. There are no lateral connections in this output layer. The connections from the input to output layer are described in terms of the connection weights (*W_ij_*). These connection weights are initialized randomly. Based on a distance measure the input vector which is closest to the weight vector is chosen to be the winner node. Only a single winner node is chosen every iteration. A popular distance measure commonly used is the Euclidean Distance (*e*) as given by Equation (3).

(3) $$e=\sqrt{\sum_{d}{\left(W_{ij,d}-V_{d}\right)}^{2}}$$

where *i*, *j* denote the location of a node in the 2 dimensional output layer of the SOM; *d* represents the dimension of the input vector V, which also corresponds to the dimension of the weight vector for a particular node *i*, *j*. When an image is used as the input, *d* corresponds to the number of pixels in the image. The weight vector corresponding to this winner node as well as those nodes in a predetermined radius around this node are then updated using Competitive learning as given in Equation (4).

(4) $${W^{\prime }}_{ij,d}=W_{ij,d}+\eta_{ij}\left(W_{ij,d}-V_{d}\right)$$

(5) $$\eta_{ij}=\eta_{max}\exp\left(-\frac{{\left(i-k\right)}^{2}+{\left(j-l\right)}^{2}}{2\sigma^{2}}\right)$$

where *W*′_*ij*,*d*_ represents the updated weights; η is the learning rate. The learning rate decays as the distance from the winning node (*k*, *l*) increases as described by Equation (5); where (*i*, *j*) denote the location of a node in the SOM output layer. σ is a parameter which represents the standard deviation of the Gaussian described in Equation (5). From Equations (4, 5) it is clear that the weight update depends on both the proximity of the weight vector to the input vector as well as the distance of the node under consideration from the winner node. This procedure is repeated for all the input vectors. The output of the SOM is given by Equation (6).

(6) $$y_{ij}=\exp\left(-\frac{\|W_{ij}-V{\|}^{2}}{2\sigma^{2}}\right)$$

where *y_ij_* represents the output for one particular node (*i*, *j*) in the output layer of the SOM; ||*|| represents the euclidean norm. On choosing the appropriate size of the output layer and the weight update parameters, a self organized structure becomes apparent on training, whereby similar input vectors are mapped onto adjacent locations in the SOM output layer. Using the SOM architecture, we simulate the development of the retinotopic map.

A filled rectangle was used as the basic shape, which served as the input to the network. Different dilations and rotations of this basic shape were presented to the network. The dilations of the rectangle were such that the aspect ratio was maintained. The aspect ratio was set to 0.1. Thus, inputs are images containing scaled and rotated rectangles of size *n* × *n*, reshaped to vectors of size *n*^2^ × 1. With this setup, we wondered if it were possible to develop a map in which rotations and dilations would be mapped onto orthogonal axes. Next we introduced a constraint on the outer boundary of the output layer in order to simulate the available surface area of V1. This is done by using a complex logarithmic transform of the vertical line through the origin in the input space. This implies from Equation (1) that the value of *x* will be 0. Therefore:

(7) u+iv=log(x+iy+a)

(8) u+iv=log(a+iy)

(9) $$u+iv=log\left(re^{i\theta }\right)$$

where

(10) $$r=\sqrt{a^{2}+y^{2}}$$

(11) $$\theta =\tan^{-1}(y/a)$$

(12) v=θ

Therefore

(13) y=atan(θ)

(14) $$r=\sqrt{a^{2}+{\left(a\tan\left(\theta \right)\right)}^{2}}$$

(15) u=log(r)

(16) $$u=log(\sqrt{a^{2}+{\left(a\tan\left(v\right)\right)}^{2}})$$

Equation (16) gives the relationship between the *u* and *v* axis in the output cortical space. The value of *a* is fixed to be 1.

**Figure 2:**
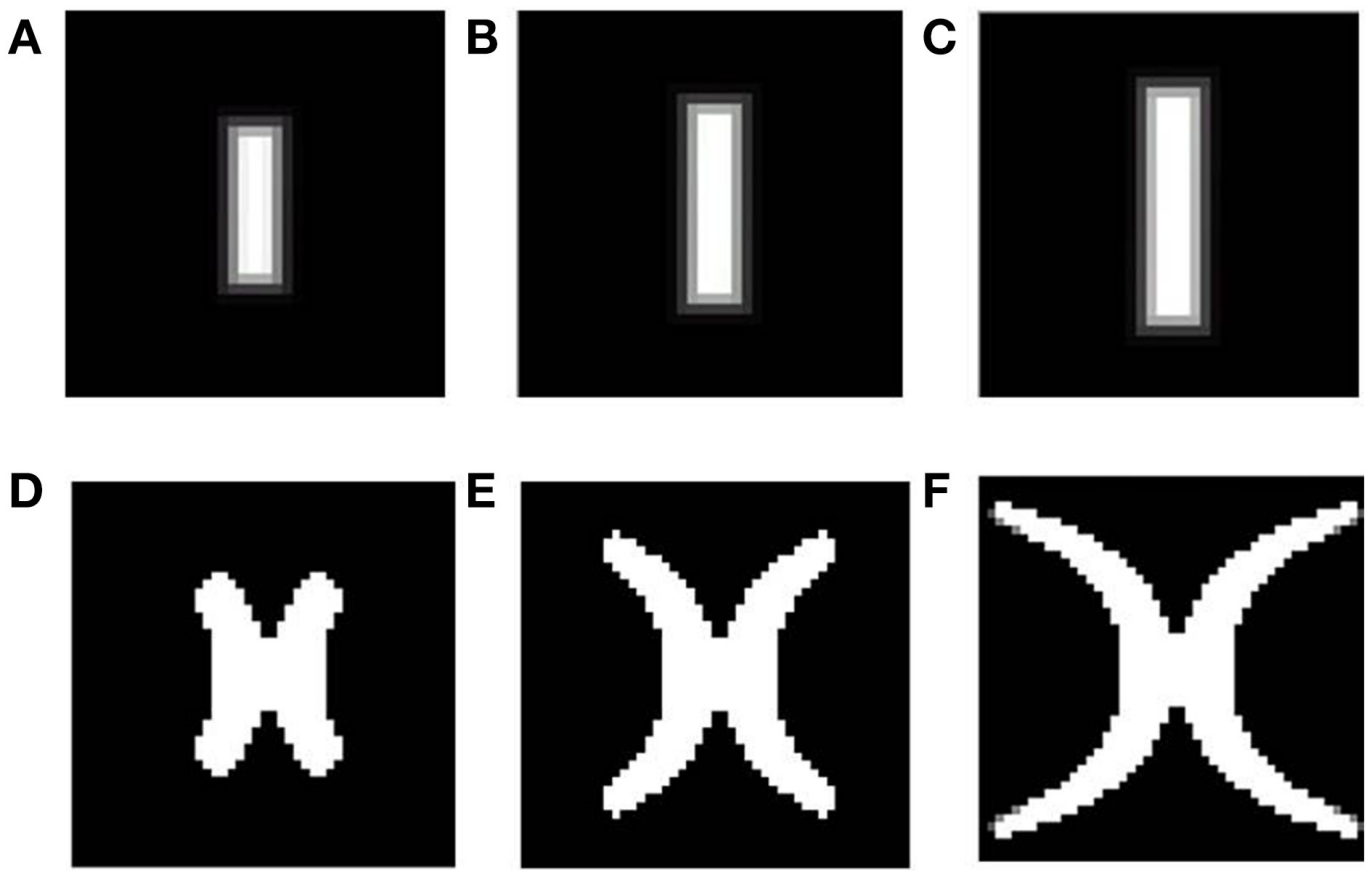
**Theoretical (complex log map) estimation (shown in D–F) of the mapping of vertical bars of increasing dilation (shown in A–C)**. As seen from the figure, the area occupied by the mapped version of the largest bar should necessarily include mapped versions of bars with the same rotation, but smaller dilation.

However, the SOM model has the following limitations in simulating the development of the retinotopic map:

- After training, since a single winner is selected for each input, each rectangular bar (of a certain rotation and dilation) is mapped onto blobs of approximately the same area, on the SOM output layer with minimal overlap. However, different dilations of a rectangular bar with a particular rotation, should in reality be mapped in such a way that, the mapped area of the more dilated bar engulfs the mapped area for each of the smaller bars of the same rotation, as shown in Figure 2. This effect is not captured by the SOM model. Thus, a bar in the visual space is mapped to a point in the cortical space; however the retinotopic map requires a “point” in the visual space to be mapped onto a “point” in the cortical space.
- Also each time the SOM is trained from scratch, a different retinotopic map is found to develop. This is due to the fact that the initial weights as well as the input vector sequence are randomized. However, from experimental studies, it is evident that the developed retinotopic map does not vary much across individuals.
- Thirdly, the SOM architecture is not entirely biologically realistic, as the selection of a winner node as well as the weight update only in a radius around this winning node, is artificially imposed.

**Figure 3:**
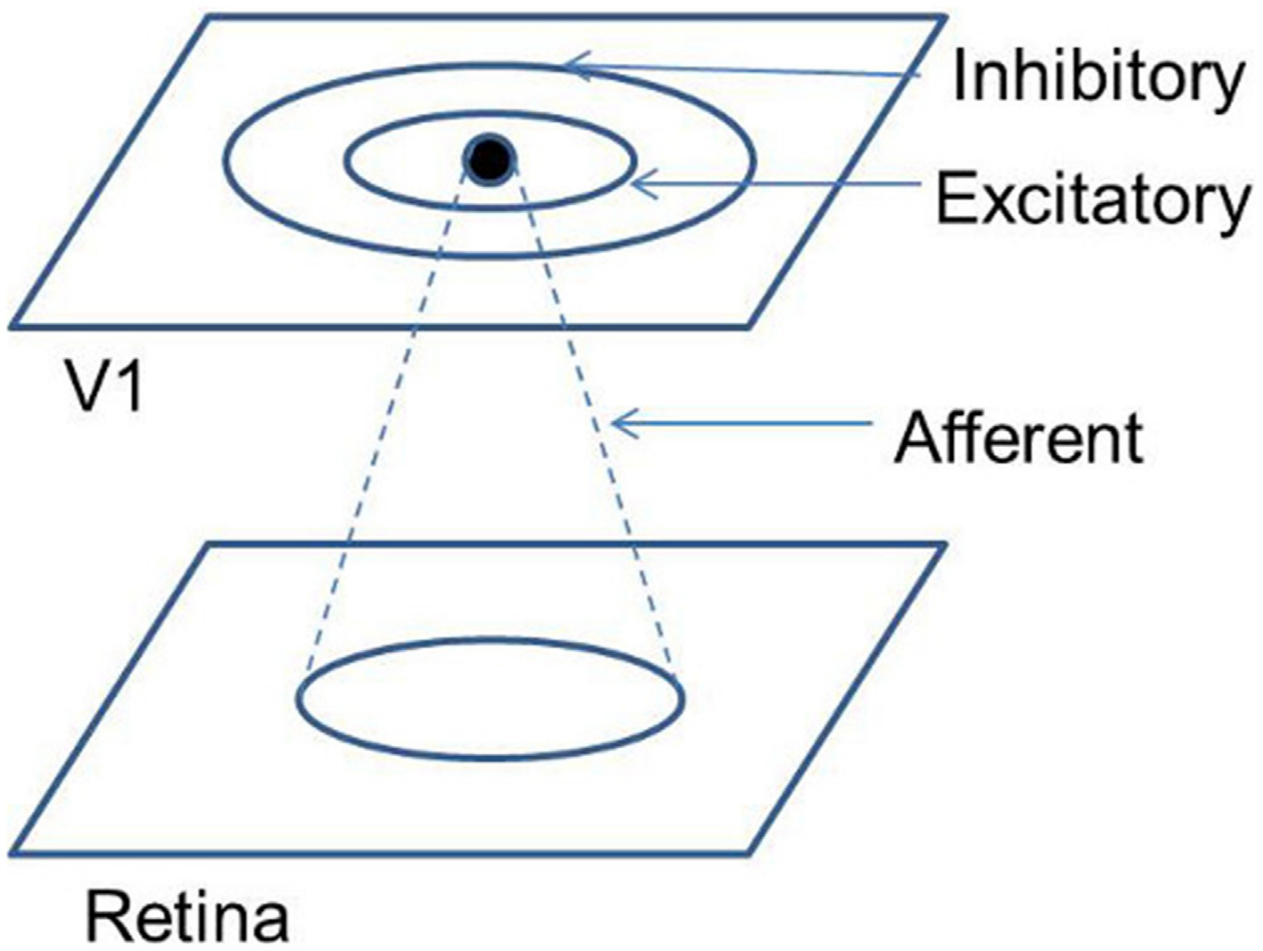
**Schematic representation of the LISSOM architecture**.

In order to overcome these limitations a variant of the SOM model known as LISSOM is utilized. Unlike the SOM, where every input node projects to every output node, in LISSOM these projections are limited to within a certain radius. The weight update also is not restricted to any particular node in LISSOM. In addition to the afferent(input to output) weights, the LISSOM architecture also include lateral(output to output) weights. This feedback mechanism is responsible for the refinement and reinforcement of the map. A schematic representation of the LISSOM architecture is provided in Figure 3. Both the afferent and lateral weights are randomly initialized within the initial radius defined. The output of a particular neuron(*y_ij_*) in the output layer, initially is dependent only on the afferent projections to that neuron as given by Equation (17).

(17) $$y_{ij}=g\left(\sum_{a,b}A_{ij,ab}x_{ab}\right)$$

where (*a*, *b*) denotes a neuron in the receptive field of the (*i*, *j*)^*th*^ neuron in the output layer, with input given as *x_ab_*; *A_ij,ab_* represents the weight from the (*a*, *b*)*^th^* neuron to the (*i*, *j*)*^th^* neuron; *g* is a piecewise approximation of the sigmoid function given as:

$$g(s)\;=\;\left\{ \begin{array}{l} 0\;:s\leq \alpha_{l}\\ (s-\alpha_{l})/(\alpha_{u}-\alpha_{l}):\alpha_{l}<s<\alpha_{u}\\ 1\;:s\geq \alpha_{u} \end{array}\right.$$

where α_*l*_ and α_*u*_ are set to 0.083, and α_*l*_ + 0.55 respectively. After this initialization the lateral connections start contributing to the output (*y_ij_*(*t*)) which depends on the output from the previous iteration (*y_ij_*(*t* − 1)). Thus, the output (*y_ij_*(*t*)) is given as:

(18) $$\begin{array}{l} y_{ij}(t)=g(p\sum_{a,b}A_{ij,ab}x_{ab}\left(t-1\right)+q\sum_{k,l}E_{ij,kl}y_{ij}\left(t-1\right)\\ \;-r\sum_{k,l}I_{ij,kl}y_{ij}\left(t-1\right)) \end{array}$$

where *p*, *q*, *r* are scaling factors; *E*_*ij*,*kl*_ is the lateral excitatory weight from neuron (*k*, *l*) to neuron (*i*, *j*) and similarly *I*_*ij*,*kl*_ is the lateral inhibitory weight from neuron (*k*, *l*) to neuron (*i*, *j*). The weight update rule is a normalized Hebbian, and is the same for afferent as well as lateral weights, as given in Equation (19).

(19) $$w_{ij,mn}(t+1)=\frac{w_{ij,mn}\left(t\right)+\eta_{ij}y_{ij}\left(t\right)P_{mn}\left(t\right)}{\sum_{mn}\left(w_{ij,mn}\left(t\right)+\eta_{ij}y_{ij}\left(t\right)P_{mn}\left(t\right)\right)}$$

where *P_mn_* is the pre-synaptic activity originating from the neuron (*m*, *n*); η is the learning rate. These learning rates can be different for each of the connections: η_*A*_, η_*E*_ and η_*I*_ are the learning rates for the afferent, excitatory and inhibitory connections respectively. With this setup in place we introduced the input images which are rotated and dilated versions of a rectangular bar. In order to perform lesion studies, we de-activated the inputs from certain portions of the input space (retinal layer) and observed the map development under these conditions.

## 3. Experiments and results

A number of simulations are performed, and each of the following subsections discuss the results which mimic certain characteristics experimentally observed in the development of the retinotopic map. These results can be summarized as follows:

- Dilations and rotations of a rectangular bar are mapped onto orthogonal axes in the SOM model; eccentricity and the meridional angle are mapped onto orthogonal axes in the LISSOM model.
- Introducing a boundary constraint makes the developed retinotopic map resemble the experimentally observed maps more closely.
- The LISSOM model overcomes the limitations of the SOM model as explained in the methods section, such that the developed map is more biologically realistic as well as stable across multiple runs.
- Lesion studies are performed, which demonstrate that a certain degree of plasticity is inherent in the development of the retinotopic map.

### 3.1. Basic SOM model

**Figure 4:**
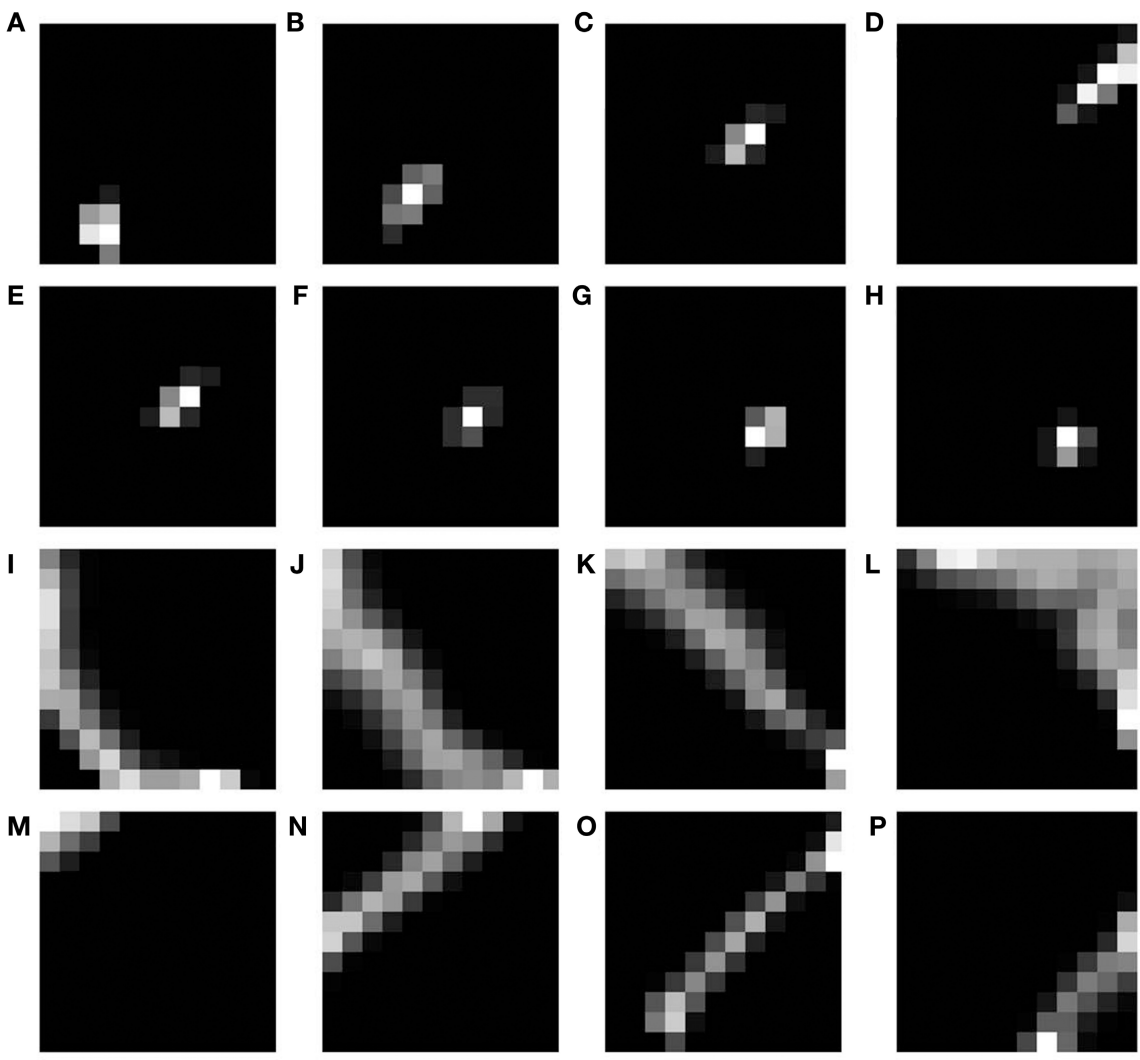
**SOM output corresponding to different inputs**. The first shows the outputs corresponding to the 9th level of dilation for **(A)** 0°, **(B)** 40°, **(C)** 80°, and **(D)** 120° as the rotational configurations respectively; Row 2 shows the outputs corresponding to 40° as the rotational configuration for the **(E)** 9th, **(F)** 10th, **(G)** 11th, and **(H)** 12th levels of dilations respectively; Row 3 shows the outputs corresponding to all possible dilations superimposed for **(I)** 0°, **(J)** 40°, **(K)** 80°, and **(L)** 120° rotations respectively; Row 4 shows the outputs corresponding to all possible rotations superimposed for the **(M)** 1st, **(N)** 5th, **(O)** 9th, and **(P)** 13th levels of dilation respectively.

**Figure 5:**
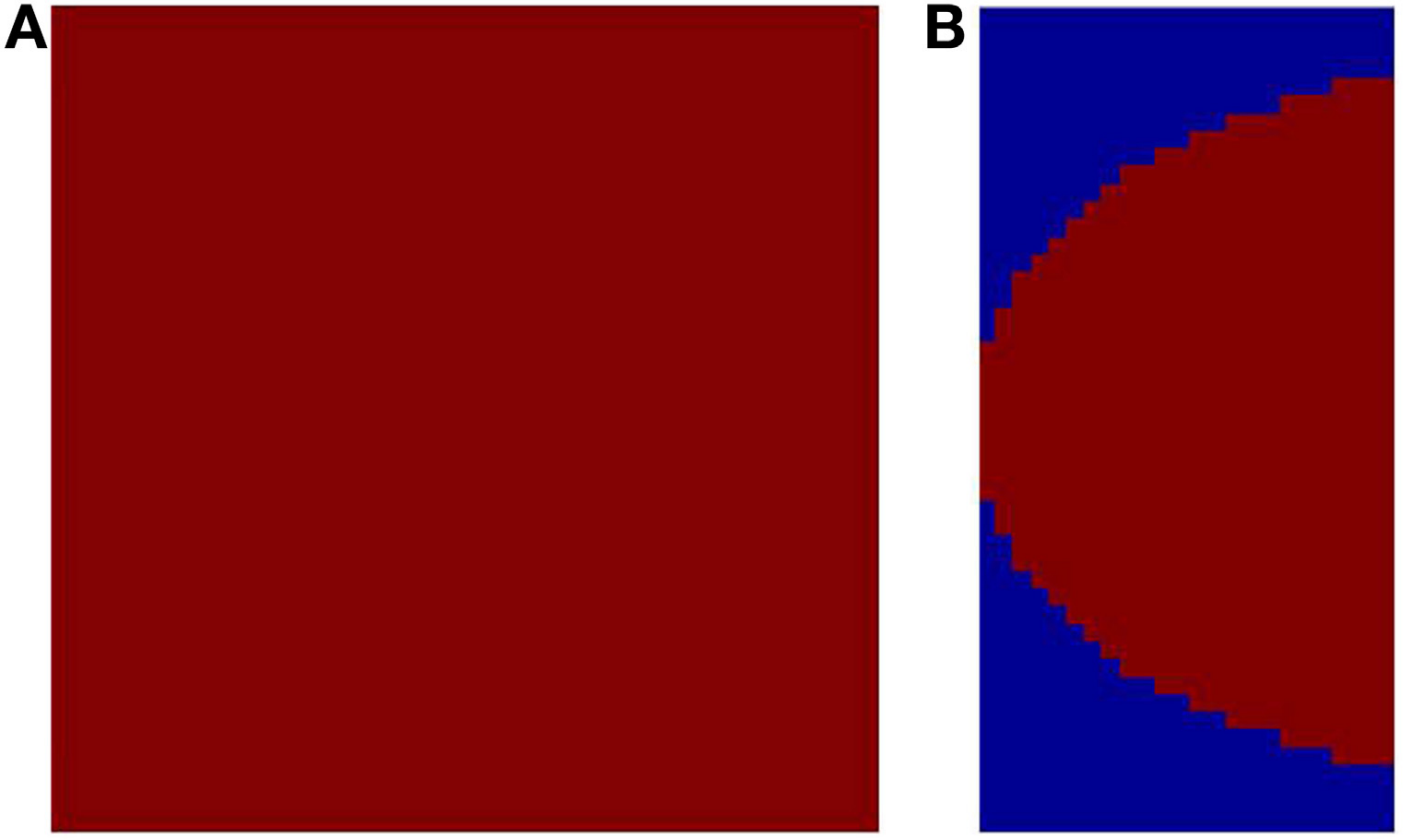
**The available output area for the SOM shown in red. (A)** without any boundary condition (12 × 12); **(B)** with a logarithmic boundary condition (24 × 48).

The basic SOM model, as described in the methods section is simulated to demonstrate that different rotations and dilations of a rectangular bar would be mapped onto orthogonal axes. The dimension of the input image is 81 × 81 pixels. The input images constituted are such that there are 9 rotational configurations (0°–180° with a step size of 20°) and 16 dilational configurations of a rectangular bar. Thus, there are in total 144 different possible configurations which serve as the inputs to the SOM. The output layer of the SOM is a square grid with 12 × 12 neurons, as shown in **Figure 5A**. The weights of the SOM are initialized randomly. The SOM is then trained for 10 epochs with the inputs presented in random order. In the SOM model convergence is normally established by observing the stability of the map after a certain number of iterations. The results shown in the paper are for 10 epochs (each epoch trains over all input vectors). The SOM model was further trained for 100 epochs and the mapping remained stable. A subset of the outputs, post training, for certain dilational and rotational configurations are shown in Figures 4A–H. Figures 4I–L shows the response of the SOM for certain rotational configurations with all possible dilations for that rotational configuration superimposed. Figures 4M–P shows the complementary response of the SOM for certain dilational configurations with all possible rotations for that dilational configuration superimposed. It is evident from this figure, that the 2 axes onto which rotation and dilation are mapped are roughly orthogonal. One important fact, which must be noted is that these outputs are for one entire training run, and will be subject to change if the SOM was once again reinitialized and retrained. This re-initialization and retraining would result in another set of orthogonal axes onto which dilations and rotations would be mapped onto, not necessarily the ones shown in the output images.

However, as mentioned earlier, the mapped version of the most dilated bar does not subsume the bars of smaller dilation, but instead, dilated versions of a bar of a particular rotation are mapped onto nearby locations in the output space (see Figures 4M–P. In order to simulate the shape of the available V1 flattened surface area, the SOM output layer was bounded by an appropriate boundary condition as described in the next section.

### 3.2. SOM model with boundary condition

**Figure 6:**
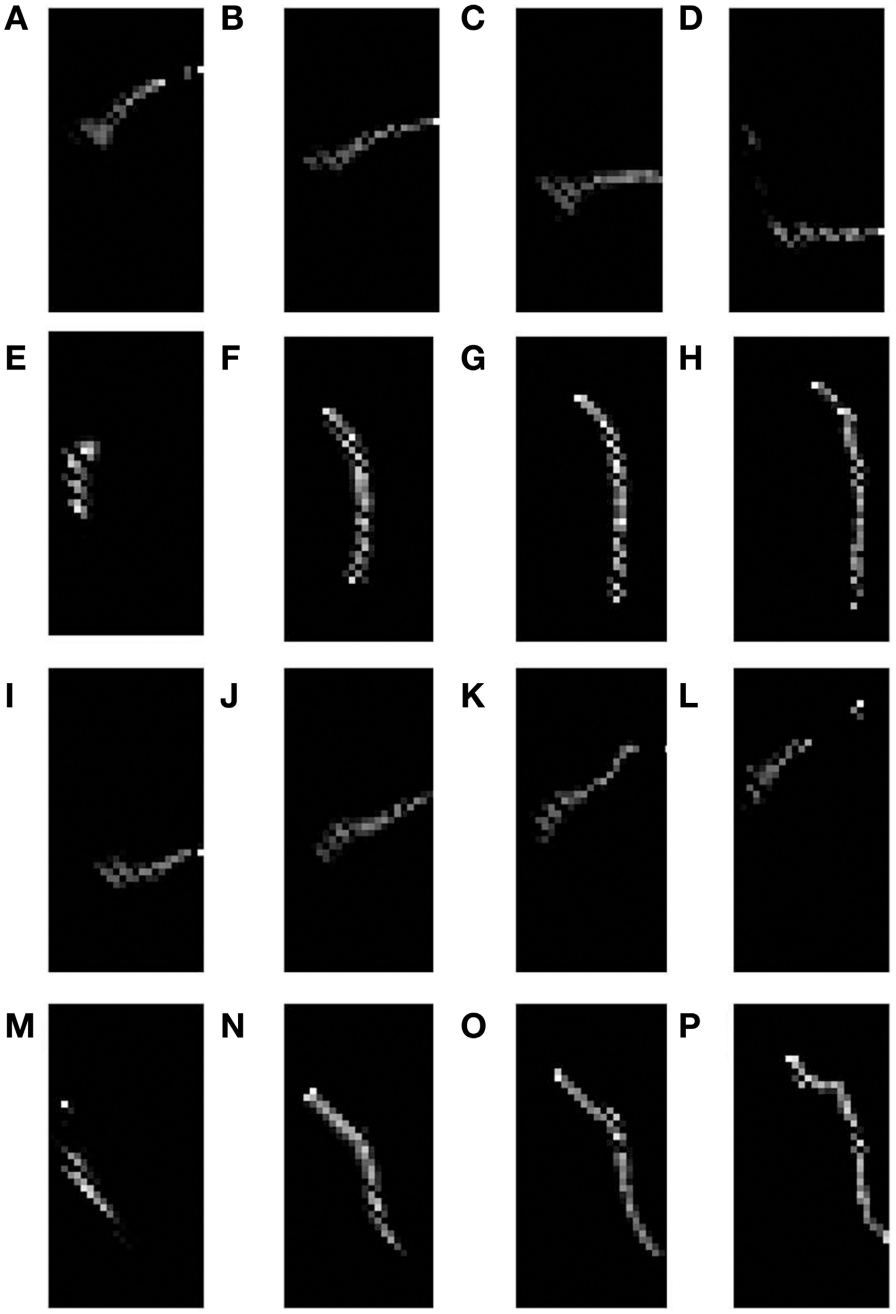
**Constrained SOM outputs superimposed corresponding to certain dilations and rotations**. The first row shows the outputs corresponding to all possible dilations superimposed for **(A)** 0°, **(B)** 40°, **(C)** 80°, and **(D)** 120° rotations respectively; Row 2 shows the outputs corresponding to all possible rotations superimposed for the **(E)** 1st, **(F)** 5th, **(G)** 9th, and **(H)** 13th levels of dilation respectively; Row 3 shows the outputs (on re-initialization of the weights and retraining the SOM) corresponding to all possible dilations superimposed for **(I)** 0°, **(J)** 40°, **(K)** 80°, and **(L)** 120° rotations respectively; Row 4 show the outputs (on re-initialization of the weights and retraining the SOM) corresponding to all possible rotations superimposed for the **(M)** 1st, **(N)** 5th, **(O)** 9th, and **(P)** 13th levels of dilation respectively.

In order to simulate a more spatially realistic version of the development of the retinotopic map, a suitable boundary condition is imposed in the SOM output layer. This is done so as to account for the shape of the available surface area in V1. A rectangular SOM with input size of 13 × 25 and output dimensions of 24 × 48 is chosen, which is then constrained. This curve is used to bound the available region of the SOM output layer, as shown in Figure 5B. The inputs to the SOM and the training paradigm remain the same as in the previous section. Figures 6A–D shows the response of the constrained SOM for certain rotational configurations with all possible dilations for that rotational configuration superimposed. Figures 6E–H shows the complementary response of the constrained SOM for certain dilational configurations with all possible rotations for that dilational configuration superimposed. Again this result is for one particular simulation of the SOM and may vary on re-initialization and retraining as shown in Figures 6I–P. Thus, this incorporation of the boundary does not cause the SOM output to be invariant across training runs.

### 3.3. LISSOM model with boundary condition

In order to overcome some of the drawbacks in the model of the development of the retinotopic map using the SOM, a LISSOM based map development is proposed. In the SOM model each input pattern (rectangular bar) is mapped onto a single point in the output space. Thus, a retinotopic mapping cannot be defined and hence the results of the SOM and LISSOM model cannot be compared. In the LISSOM model we consider 2 layers: the input retinal layer with dimensions of 25 × 25 neurons, and the output V1 layer with dimensions of 48 × 48 neurons. The 48 × 48 output layer is constrained using a complex logarithmic transform of the vertical line through the origin in the input space, as in the previous section. The value of *a* is fixed to be 1. The Topographica Simulator (Bednar, 2009) is used to perform these simulations. The parameters of the model are given in Table 1.

**Table 1:** **Parameter values chosen in the LISSOM model for the simulation of the retinotopic map**.

**Parameter**	**Value**
*p*	1.5
*q*	1.1
*r*	1.1
η_*A*_	0.3
η_*E*_	0.25
η_*I*_ (inital)	0.25
η_*I*_ (after 500 iterations)	0.5
*rad_A_*	1 (25)
*rad_E_*	0.03 (1.44)
*rad_I_*	0.55 (26.4)

*The radii terms contain the radii in grid units for a 25 × 25 input and 48 × 48 output LISSOM in brackets*.

The central 4° of visual space, which maps onto 27% of the primary visual cortical space (Adams and Horton, 2003) is simulated using the LISSOM architecture. The cortical distance from the apex (fovea) to the point which maps 4° eccentricity, 0° meridional angle is approximately 9 mm (Adams and Horton, 2003). The maximum extent of lateral connections in V1 is roughly 8 mm (Gilbert and Li, 2012). In the primary visual cortex the long-range inter-columnar connections are predominantly inhibitory in effect for high contrast input stimuli, due to local inhibitory inter-neurons (Hirsch and Gilbert, 1991; Weliky et al., 1995). Hence in the LISSOM model the long range lateral connection are inhibitory and have a maximum radius of *rad_I_* set to 0.55 which corresponds to a radius of 26.4 in a 48 × 48 output LISSOM, which in cortical length corresponds to 9.9 mm. This roughly matches the known radius of lateral connections in V1. There also exist short range (≤0.5 mm) lateral connections in V1 (Stettler et al., 2002). The short range connections in V1 could be both excitatory as well as inhibitory (Kisvarday et al., 1997). The short range inhibitory connections are considered a subset of the larger radius inhibitory connections specified in the LISSOM model. The short range excitatory connections are modeled in the LISSOM having a maximum extent of *rad_E_* = 0.03 which corresponds to radius of 1.44 in a 48 × 48 output LISSOM, which in cortical length corresponds to 0.54 mm. Thus, each neuron in the V1 layer of LISSOM excites up to 8 of its neighboring neurons.

**Figure 7:**
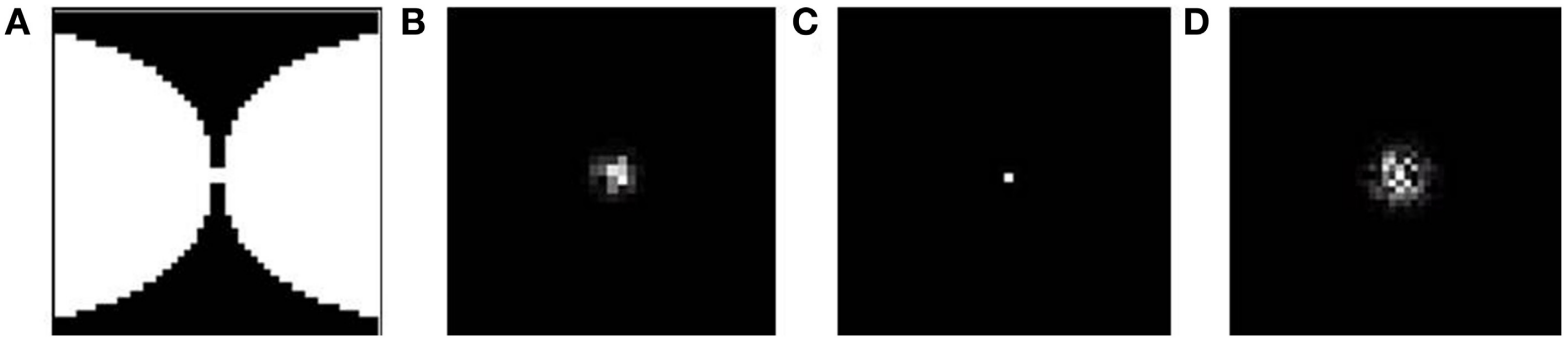
**Boundary and initial conditions for the LISSOM model. (A)** shows the available neurons in the output layer of the LISSOM in white, the boundary condition is set as per Equation (16); **(B–D)** represent the initial weights of afferent, excitatory and inhibitory connections respectively.

Initially the afferent, excitatory and inhibitory connections have a small radius and corresponding weights in that radius as shown in Figures 7B–D, and are allowed to grow with some bounding values. These bounding values for the radius of afferent, excitatory and inhibitory connections are denoted as *rad_A_*, *rad_E_*, and *rad_I_* and are given in Table 1. In order for the map to develop as required it is essential that *rad_A_*, and *p* are greater than *rad_I_*, and *r* respectively so as to allow the spread of the map in the direction allowed by the outer logarithmic boundary constraint (see Equation 16) as shown in Figure 7A. In order to train the map, as mentioned earlier rectangular bars of varying dilations and rotations are utilized. During the testing stage, that is to demonstrate that the retinotopic map indeed develops, a point in the input space should be mapped on to a point in the output space. Thus, in the testing stage single point inputs are used to construct the map.

**Figure 8:**
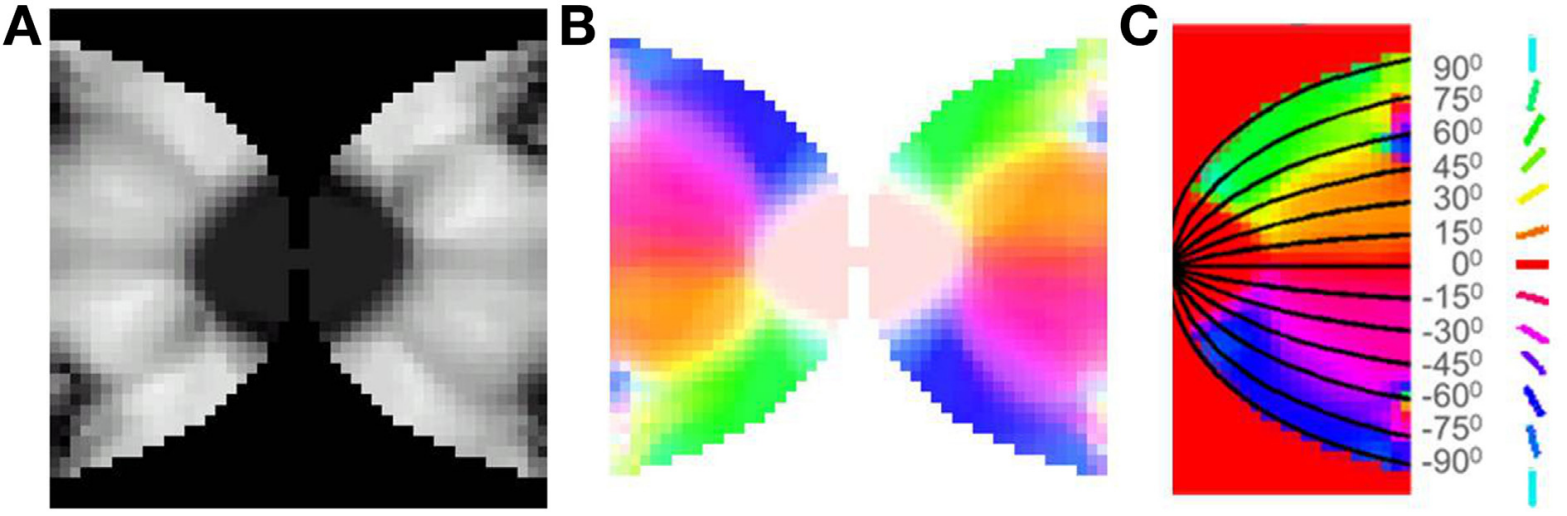
**V1 retinotopic map. (A)** Meridional angle selectivity (gray scale coded: black represents least selective portion, while white the most selective one); **(B)** Meridional angle selectivity and preference combined; **(C)** Meridional angle preference (color coded), superimposed with the complex logarithmic map for validation.

**Figure 9:**
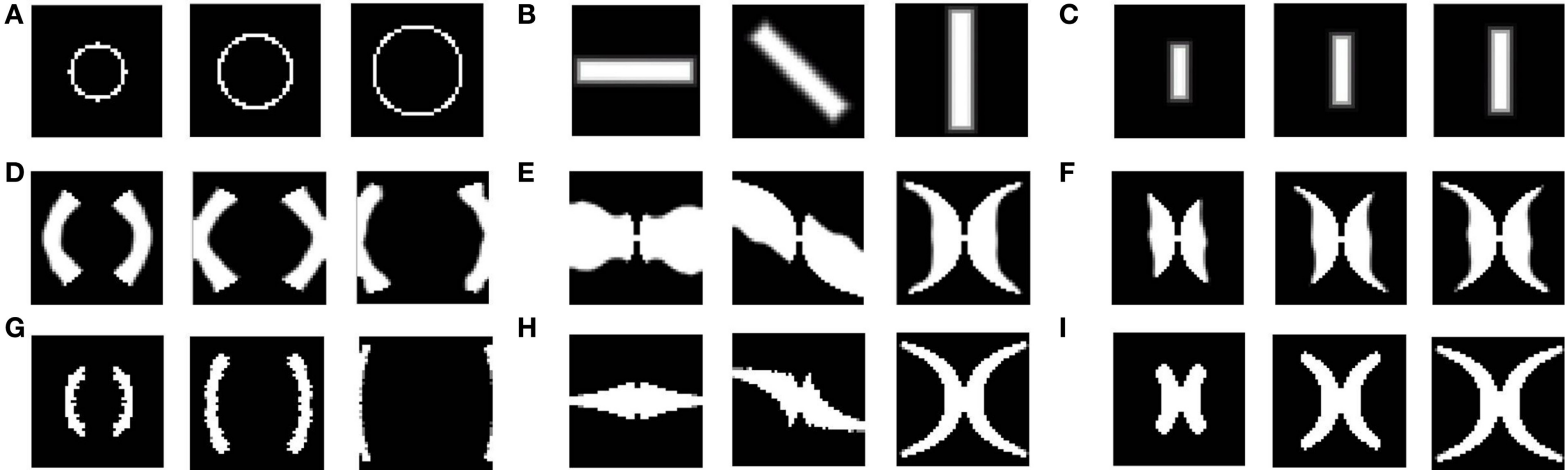
**LISSOM outputs corresponding to different inputs, post training. (A)** shows circular rings of increasing radii given as input; **(B)** shows rectangular bars of 0°, 45°, and 90° given as input; **(C)** shows rectangular bars of increasing dilation 0.6, 0.8, and 1 given as input; **(D–F)** shows the corresponding LISSOM outputs for the given inputs; **(G–I)** shows the corresponding complex log map transformation for the given inputs.

After training, it may be observed in the developed map that the meridional angle is mapped along the y-axis (see Figure 8C); while the eccentricity is mapped along the x-axis (see Figures 9A,D,G), an organization that bears strong resemblance to the complex logarithmic map. It may be observed that the central region is not very selective for the meridional angle, a fact attested by the selectivity plot (see Figures 8A,B). In reality this central region should be a single point at which all the mapped meridional angles converge at the apex of the V1. However, due to the minimum possible size of the rectangular bar, so as to still maintain the aspect ratio, as well as to ensure a smooth change in the learnt output of the LISSOM, this central region which is non-selective to meridional angles is slightly larger in the model. This is an artifact of the simulation. The region outside the logarithmic boundary in Figure 8C is color coded as red by default, however this region is unresponsive to any of the input patterns as seen in Figure 8B.

After training, the map developed for varying eccentricities, is also qualitatively similar to the theoretical estimation as given by the complex log map, shown in Figures 9A,D,G. The one major difference is that the theoretical estimate is on the log scale where as the LISSOM outputs are on a linear scale along the eccentricity axis. The logarithmic nature of eccentricity of the retinotopic map, as mentioned earlier, is a consequence of the exponential decay of the density of RGCs from the fovea, radially outward (Wässle et al., 1990). The logarithmic transformation of eccentricity in the retinotopic map could be approximated to a linear transformation of the same. Thus, in all results where eccentricity is being mapped a linear ratio is maintained in dilation between different inputs used in the LISSOM model for map development; whereas the same exponential ratio is maintained in order to define the inputs for the complex logarithmic map used for validation.

**Figure 10:**
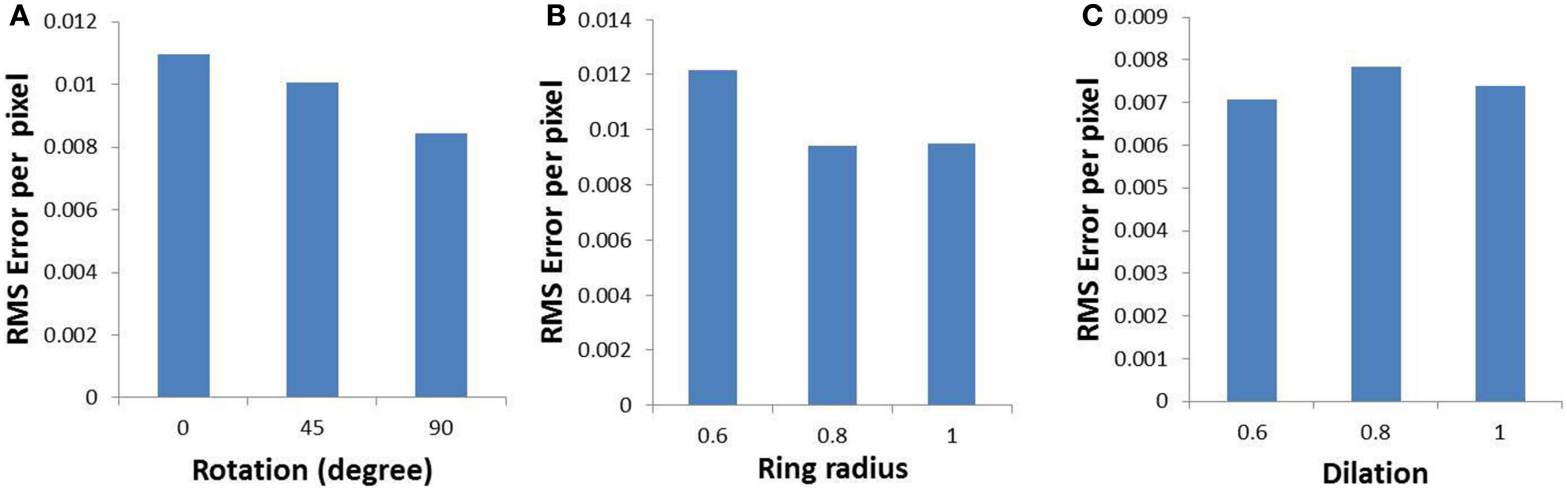
**Root mean square error (per pixel) between the developed map and the complex log map for (A) a rectangular bar at different rotational configurations, (B) circular rings of increasing dilation, and (C) a rectangular bar at different dilations, given as inputs to the LISSOM**.

In order to validate the model, the output of the model is compared with the output of the complex log map and the pixel by pixel RMS error per pixel is calculated. This RMS error per pixel is computed for 3 different input types: circular rings of increasing dilation (see Figures 9A,D,G), a rectangular bar with fixed dilation but changing rotation (see Figures 9B,E,H), a rectangular bar with fixed rotation but changing dilation (see Figures 9C,F,I). The low RMS error demonstrates a reasonable match, as can also be visually ascertained. As mentioned earlier a linear ratio is maintained in dilation between different inputs used in the LISSOM model for map development; whereas the same exponential ratio is maintained in order to define the inputs for the complex logarithmic map used for validation. The RMS error between the model output and the complex log map estimate is shown in Figure 10.

**Figure 11:**
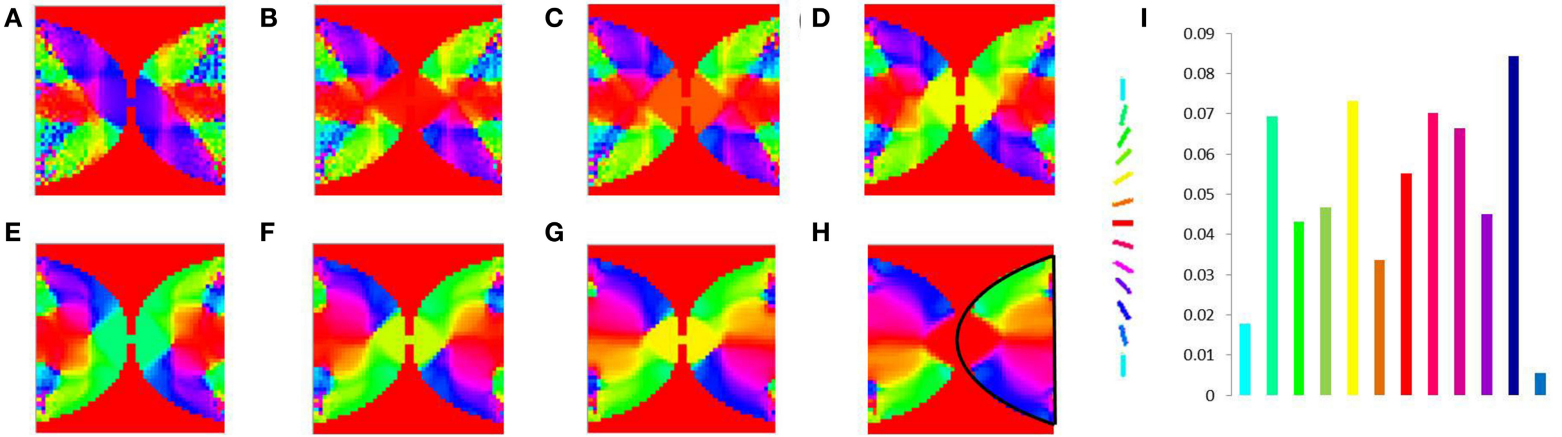
**V1 map development**. Meridional angle preference (color coded) at **(A)** 200, **(B)** 300, **(C)** 400, **(D)** 500, **(E)** 600, **(F)** 700, **(G)** 800, **(H)** 900 iterations respectively, **(I)** shows the histogram of the pixels based on their meridional angles present within the black boundary in **(H)**. The initial retinotopy is shown in Supplementary Material Figure 3.

**Figure 12:**
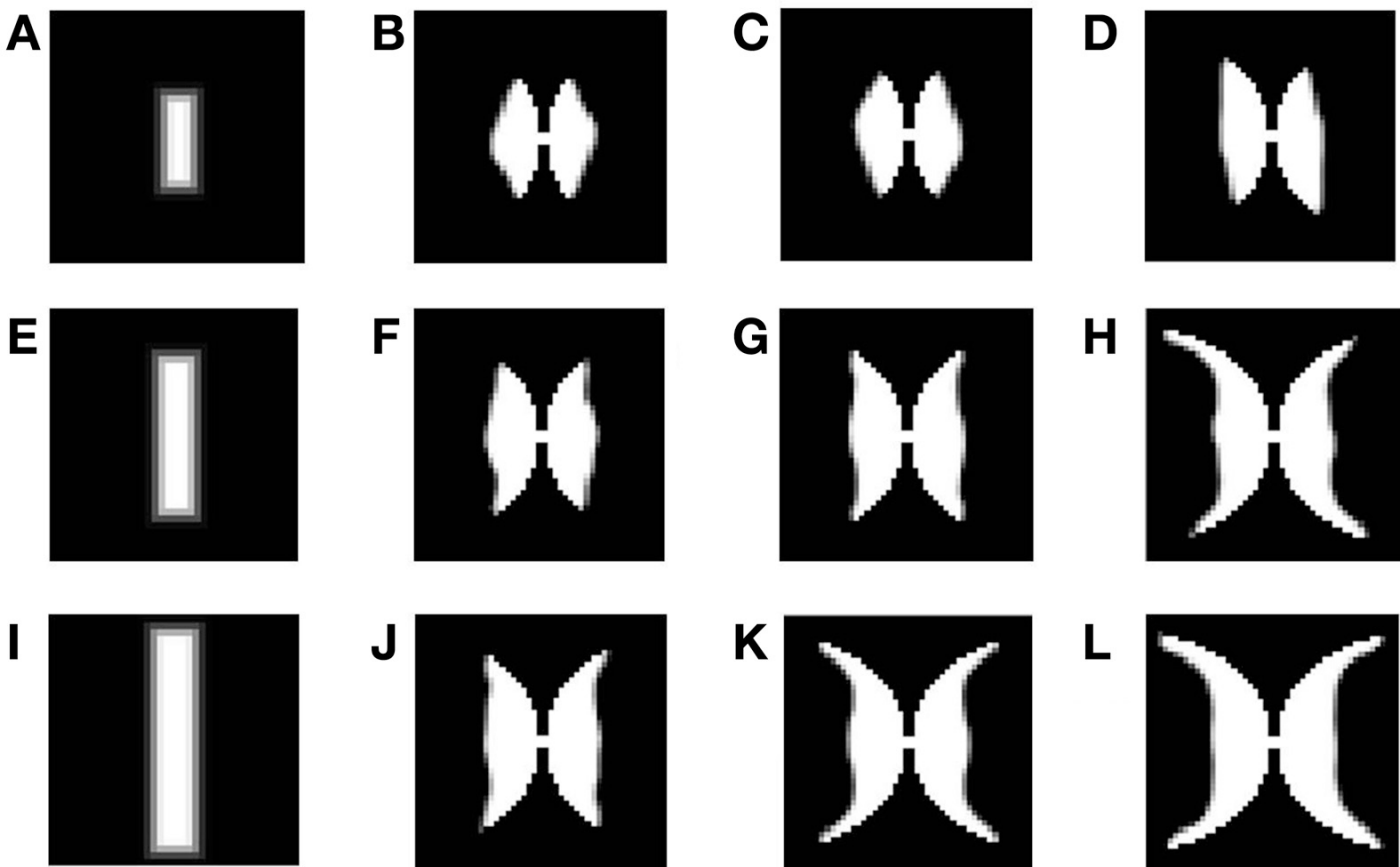
**Demonstration of the development of the map using LISSOM across iterations, considering vertical rectangular bars of different dilations as inputs**. **(A,E,I)** are the inputs given to the LISSOM; **(B–D)**; **(F–H)**; **(J–L)** are the corresponding outputs of the LISSOM after 200, 400, 600 iterations respectively.

**Figure 13:**
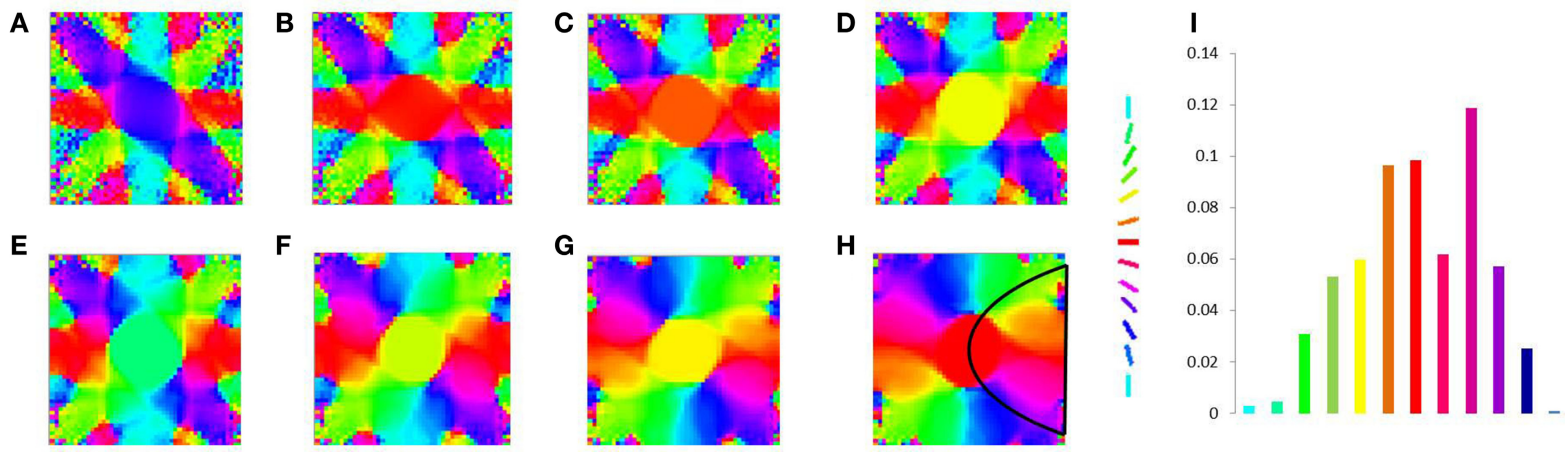
**V1 map development without any boundary condition**. Meridional angle preference (color coded) at **(A)** 200, **(B)** 300, **(C)** 400, **(D)** 500, **(E)** 600, **(F)** 700, **(G)** 800, **(H)** 900 iterations respectively, **(I)** shows the histogram of the pixels based on their meridional angles present within the black boundary in **(H)**.

In order to demonstrate the map formation, we simulate the development of the map for meridional angle across iterations as shown in Figure 11. Similarly the development of the map for eccentricities is demonstrated by using a vertical bar of varying dilation and its corresponding LISSOM output is shown in Figure 12. In one sense the boundary condition for the map, guides the formation of the retinotopic map in the model. If we changed this boundary condition, a very different retinotopic map would be developed. To demonstrate this we simulated the LISSOM with exactly the same parameters, but without any boundary constraint. The map developed is shown in Figure 13. In order to quantify this difference we plot a histogram of the pixels based on the color code of their meridional angles present in the developed map within the same cortical area (shown by the black boundary in Figures 11H, 13H. The histograms correspond to the area occupied by each meridional angle in the map developed. As seen from Figures 11I, 13I the histogram for the map developed without the boundary condition allocates more area to the central meridional angles compared to the map developed with the boundary condition.

**Figure 14:**
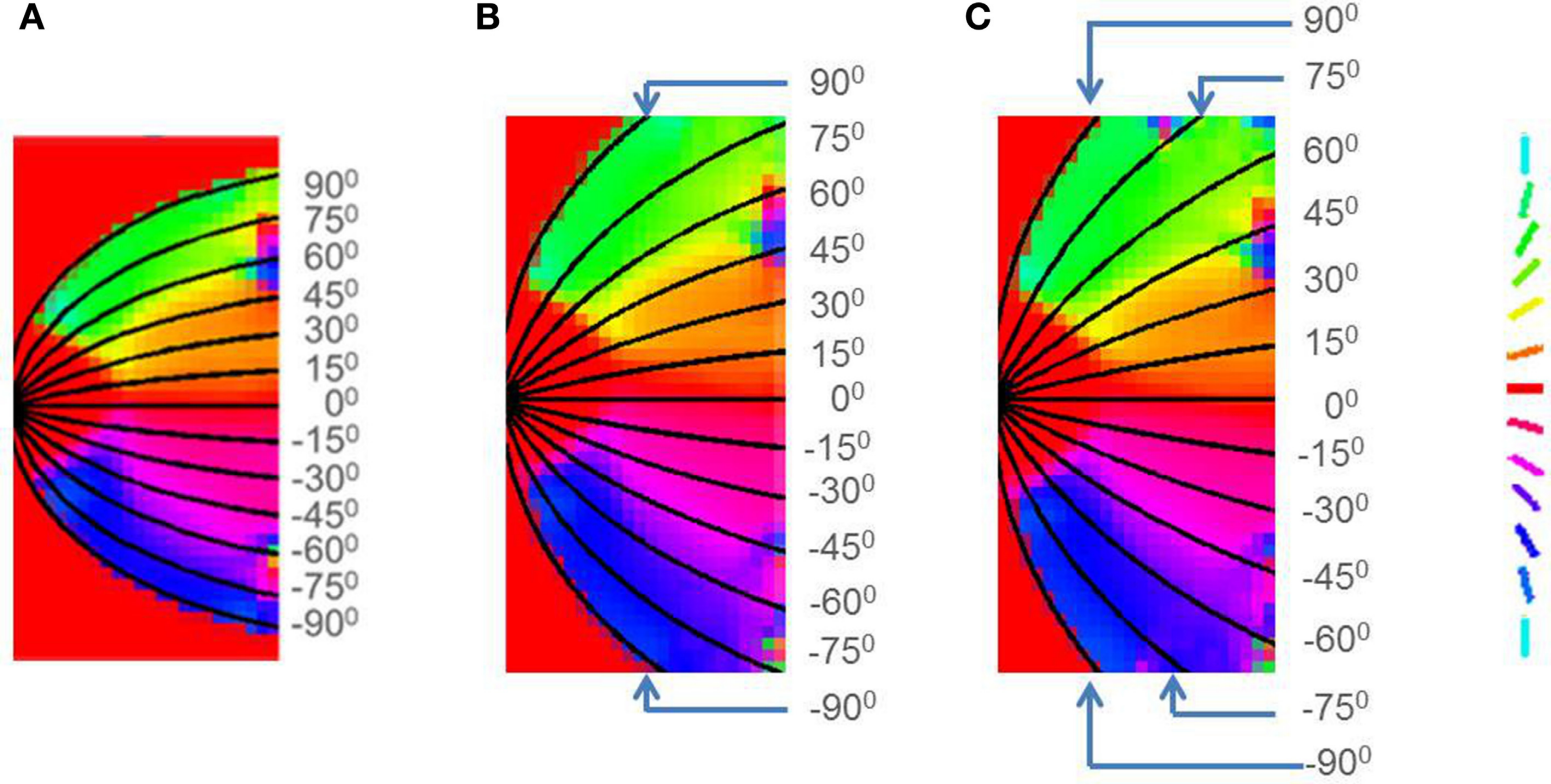
**Retinotopic map developed with different boundary conditions imposed by changing the value of *a* in Equation (16) for (A) Squirrel monkey with *a* = 1, (B) Owl monkey with *a* = 2.5, (C) Cat with *a* = 4**.

This result has been experimentally observed: if the V1 cortical arealization is itself altered, the map formed would also be altered (O'Leary et al., 2007). This can also be demonstrated by developing the retinotopic map for different species, by changing the boundary conditions imposed, by varying the value of *a* in Equation (16) as shown in Figure 14.

A number of additional simulations are performed to illuminate the various factors which lead to the emergence of the retinotopic map as described by the LISSOM mechanisms. The simulation results along with a brief description on what is inferred from each of these simulations are included in the supplementary material. These additional results can be summarized as follows:

- It is the pattern of the objects (i.e., its rotations and dilations) in the inputs that is necessary for the map formation rather than the shape of the object itself.
- The rough retinotopy imposed initially in the LISSOM architecture is not sufficient for the final map formation.
- Each of the 3 connection types: afferent, lateral excitatory, and lateral inhibitory are vital for the final map formation.
- Decreasing the maximum inhibitory radius (*rad_I_*) results in discontinuities in the final map formed.
- Increasing the maximum excitatory radius (*rad_E_*) results in an additional (beyond requirement) spread in the final map formed.

### 3.4. Lesion studies with LISSOM

**Figure 15:**
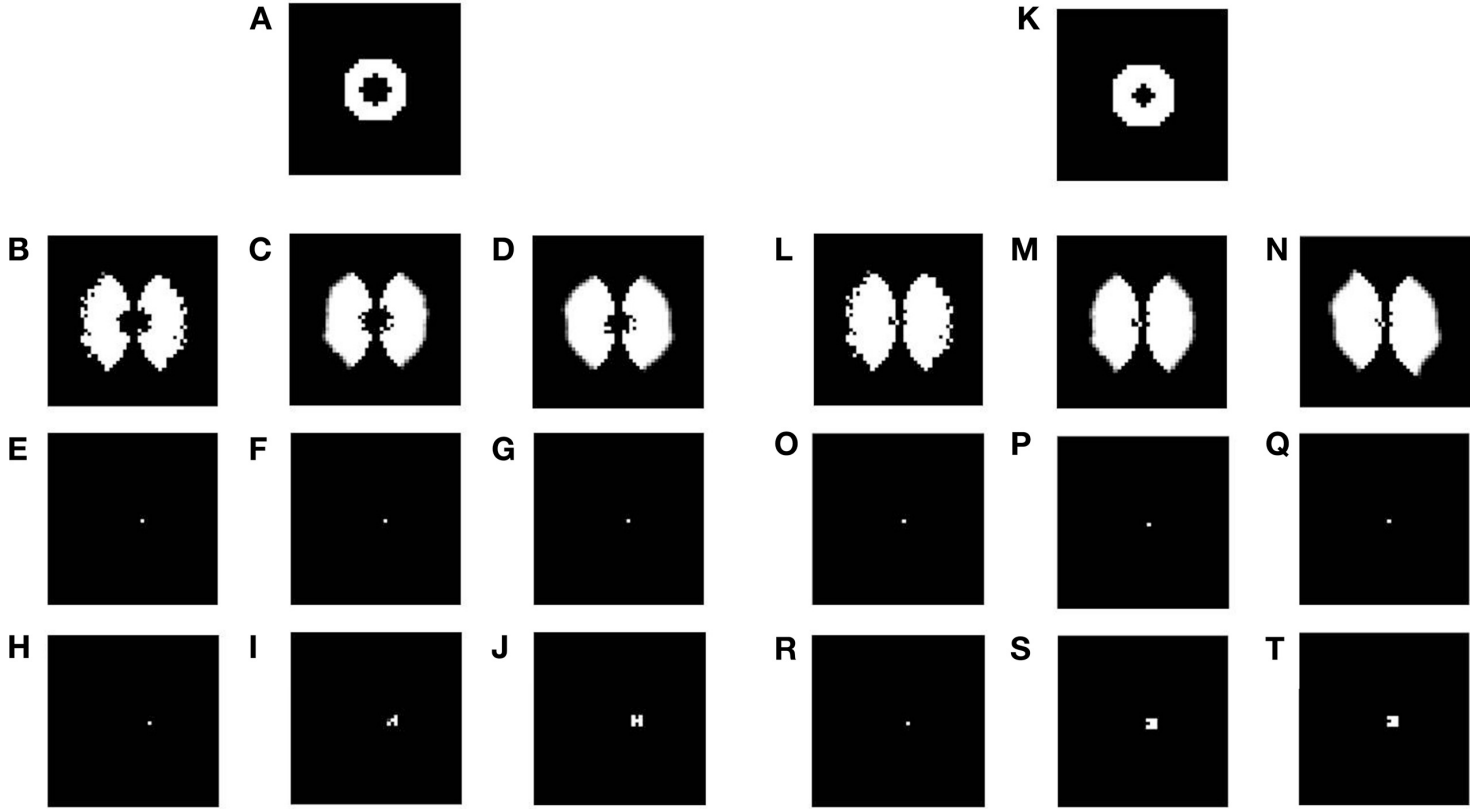
**Plasticity in excitatory lateral connections in V1 simulated on introducing retinal lesions**. **(A,K)** show a circular disc given as the input to the LISSOM with the retinal layer having a circular lesion with its center; **(B,L)** show the corresponding output activity of the LISSOM before training; **(C,M)** show the corresponding output activity of the LISSOM after 100 iterations; **(D,N)** show the corresponding output activity of the LISSOM after 200 iterations; **(E–G)** show the excitatory connection weights for a neuron (24, 26) which is inside the LPZ across iterations (0,100,200) on giving input as **(A)**; whereas **(H–J)** show the excitatory connection weights for a neuron (24, 28) which is outside the LPZ across iterations (0,100,200) on giving input as **(A)**; **(O–Q)** show the excitatory connection weights for a neuron (24, 25) which is inside the LPZ across iterations (0,100,200) on giving input as **(K)**; whereas **(R–T)** show the excitatory connection weights for a neuron (24, 26) which is outside the LPZ across iterations (0,100,200) on giving input as **(K)**.

In the previous section the plasticity observed in the map was predominantly in the afferent connections. In order to demonstrate the plasticity in the lateral connections, we simulate the same model, but with lesions. Retinal lesions are introduced into the input retinal layer of the LISSOM and, map development under these conditions are observed. The size of the retinal lesions play a crucial role in whether or not the LPZ in the output cortical layer will show any response activity. For testing the extent of plasticity a circular disc is given as an input and the activity in the output layer is observed. The center of this disc where there is no input activity represents the retinal lesion. The neurons in the output layer which receive afferent projections from the lesioned area (LPZ) will initially shown reduced activity. However, due to the excitatory neurons in V1, the LPZ will gradually shrink in size (see Figures 15A–J). If the entire LPZ is within the range of the excitatory laterals, the LPZ may in fact disappear (see Figures 15K–T). As seen from Figures 15H–J,R–T there is an increase in the radius of excitatory lateral connections just outside the LPZ; however there is no such increase for neurons within the LPZ as shown in Figures 15E–G,O–Q. These results are similar to those experimentally observed (Kaas et al., 1990; Schmid et al., 1996; Eysel et al., 1999), where there is activity observed even in the LPZ shortly after lesioning. As speculated (Gilbert and Li, 2012) the simulations seem to verify that this kind of plasticity is primarily due to lateral connections in V1.

## 4. Discussion

The mapping of the visual space onto the primary visual cortex in primates is one of the earliest topographic maps discovered in the cortex. A function which empirically fits this transformation of a point in the visual space to a point in the cortical space was proposed (Schwartz, 1977), namely the complex logarithmic function. Although there have been attempts to refine this mapping function for a better fit (Balasubramanian et al., 2002; Schira et al., 2010), there are few computational models which simulate the map formation itself. The computational models that simulate the development of the retinotopic map in the primary visual cortex could be broadly classified as chemo-specific, spontaneous neural activity dependent and, stimulus dependent (Goodhill, 2007). Chemo-specific models are those which simulate the development of the map based on certain molecular gradients expressed (Gierer and Meinhardt, 1972; Prestige and Willshaw, 1975; Brown et al., 2000; Koulakov and Tsigankov, 2004), whereas activity dependent models require correlated activity for the map development (von der Malsburg and Willshaw, 1976; Linsker, 1986). It is speculated that both chemo-specific and spontaneous neural activity dependent mechanisms are required for the initial development of the retinotopic map. Stimulus dependent development is initiated post eye opening, whereby the primary visual cortex circuitry is refined to form more precise retinotopic maps (Smith and Trachtenberg, 2007). However, the models which do simulate stimulus dependent retinotopic development do not simulate the global nature of the retinotopic map (Swindale, 2000).

It is thus evident that all three mechanisms based on: molecular gradients, spontaneous retinal waves and visual stimuli are involved in the retinotopic map formation. However, the final refinement of the map appears to be visual stimuli dependent. In the simulations performed we assume an initial rough retinotopy which is almost identical to the retinotopy present in the retina, i.e., eccentricity and meridional angle are mapped onto the polar co-ordinates in the cortical space. This initial topography is assumed to be provided by chemical gradients or the spontaneous retinal waves. The LGNd layer which acts as a transmitting layer is not simulated. The internal connections in V1 are also roughly approximated in the later simulations. Thus, in this paper, we demonstrate that given a rough topography preserving mapping from the retina via the LGNd to the V1 and given the appropriate training criteria, a retinotopic map resembling experimental findings could be developed, by a stimulus driven activity dependent mechanism.

One of the insights gained from the retinotopic map in primates is that a complex logarithmic transformation results in rotation and dilation variation in the input visual space to be transformed to a shift (translational) variation in the output space along 2 distinct axes (Schwartz, 1977). Now if we considered the image of any object on the retina to be represented by a 2-dimensional matrix, rotations and dilations could be considered to be primary transformations of this matrix. This led us to speculate whether rotations and dilations of a particular object (in our case a rectangular bar) would self-organize in such a way that these would be mapped onto distinct axes. The initial simulations demonstrate the validity of this hypothesis: indeed rotations and dilations are mapped onto distinct axes. However, there are quite a few drawbacks in the SOM model, the major one being that the entire rectangular bar is mapped on to a single point in the SOM output space, instead of the constituent points of the rectangular bar being mapped onto their corresponding transformed points in the output space. Thus, dilations and rotations are mapped onto orthogonal axes instead of the eccentricity and meridional angle. Also in the SOM as the first winner node selection is random, since we initialize the weights randomly, every time the SOM algorithm runs a different configuration of the SOM output is seen. This is not the case in the LISSOM model, since each node in the LISSOM receives input only from a small radius of neurons in the retinal layer initially. This radius is allowed to grow with time.

Thus, the LISSOM model was found to be more suitable for our purpose. The idea here is to train the LISSOM model on dilations and rotations of simple patterns, and test it as a point-to-point retinotopic map with point inputs. The LISSOM model is then simulated and it is observed that the output retinotopic map developed is more accurate and biologically plausible than the one obtained with the SOM model. It may be observed in the developed map that eccentricity is mapped along the horizontal axis, while the meridional angle is mapped along the vertical axis, an organization that bears strong resemblance to the complex logarithmic map. The simulated lesion studies hint at the importance of lateral excitatory feedback. As seen from the simulation results the amount of plasticity possible is restricted by the radius of lateral excitatory connections. In the adult V1 this is the only form of plasticity possible since the afferent connections are already refined.

An additional insight we gain from the LISSOM simulations, is that the boundary condition imposed on the available area of V1, plays an important role in the map development. Recent studies have demonstrated that retinal waves drive correlated patterned activity in the superior colliculus as well as V1, during the developmental period (Ackman et al., 2012). This has led to speculation that activity dependent mechanisms could be involved in the topography preserving nature of the retinotopic map. However, this correlated activity, without the appropriate boundary condition, would lead to the development of a totally different retinotopic organization (O'Leary et al., 2007) as shown in Figure 13 and Figure 14. We speculate that the available V1 area, is in part responsible for the complex logarithmic nature of the retinotopic map.

The LISSOM model also elucidates the appropriate radii of afferent and lateral connections required for the map development. The amount of spread possible in the map is a direct consequence of the radius of afferent connections. This afferent radius then dictates the radius of inhibitory lateral connections required for the map development. If too small an inhibitory radius is chosen, it would result in discontinuities in the output activity. If too large an inhibitory radius is chosen, the spread in the output map would be hampered. In the adult V1, both the afferent and lateral connection projections are too short range, to account for such large scale changes in the map as shown in the LISSOM simulations. Thus, the map in the adult V1 is robust to changes in the visual input and only small variations are possible once the map is fully developed. These changes are predominantly due to plasticity in the lateral connections as mentioned earlier.

This raises the possibility of the training regime described in this paper being used to develop the correct retinotopic map if in case the map developed is abnormal as in the case of albinism. The normal afferent visual fibers from the retina are disrupted in albinism, where the line of decussation is moved into the temporal retina and as a result the subsequent map formed in V1 is improper (Hoffmann et al., 2003). Even if it were possible to redirect the afferent fibers in such a way that would resemble the normal projections to V1, the map formation is not guaranteed. One way to facilitate this formation would be to reestablish a rough arrangement of the axon terminals on to neurons in V1, followed by the training regime of an image at various dilation and rotation given as visual inputs to the subject as described in this paper. A prerequisite for this kind of map formation is an induced plasticity in the afferent and lateral connections.

One of the limitations of the model is that the logarithmic nature of the transformation of eccentricity is not captured. This is due to the fact that the exponential decay of RGCs neurons radially outward is not incorporated in the model. Secondly although the map developed captures the retinotopic nature of V1, the other maps in V1 are not seen in the model. This is because of the coarse nature of the map. A more detailed model which incorporates both the logarithmic nature as well as the multiple maps in V1 would be an interesting future perspective.

## Author contributions

Ryan T. Philips, Computational model development, analysis and manuscript preparation; V. Srinivasa Chakravarthy, Computational model development, analysis and manuscript preparation.

### Conflict of interest statement

The authors declare that the research was conducted in the absence of any commercial or financial relationships that could be construed as a potential conflict of interest.

## Supplementary material

The source code required to replicate the results of the simulations performed in this paper would be made available on personal request to the authors.
