# Supplementary material for: The mapping of eccentricity and meridional angle onto orthogonal axes in the primary visual cortex: an activity-dependent developmental model
Source: Front Comput Neurosci. 2015 Jan 29;9:3. doi: 10.3389/fncom.2015.00003 (PMC4310300; doi:10.3389/fncom.2015.00003)
Supplement: Supplementary file 1 [file DataSheet1.PDF]

Frontiers in Computational Neuroscience

Research Article
29 October 2014

Supplementary Material: The mapping of eccentricity and meridional angle onto orthogonal axes in the primary visual cortex: An activity-dependent developmental model

Ryan Thomas Philips [1] **and V. Srinivasa Chakravarthy** [1,*]

[1] *Computational Neuroscience Laboratory, Indian Institute of Technology Madras, Department of Biotechnology, Chennai, India*

Correspondence*:
V. Srinivasa Chakravarthy
Computational Neuroscience Laboratory, Indian Institute of Technology Madras,
Department of Biotechnology, Chennai, 600036, India, schakra@iitm.ac.in

1 SUPPLEMENTARY TABLES AND FIGURES

A number of simulations are performed to illuminate the various factors which lead to the emergence of the retinotopic map as described by the LISSOM mechanisms. In each of these simulations all factors other than the ones mentioned specifically to be changed are maintained as the default ones.

The nature of the inputs given to the LISSOM play a crucial role with regard to whether an accurate retinotopic map develops. It is essential that inputs given are dilations and rotation of a particular template shape. Thus for example, if dilated and rotated versions of an ellipse were used instead of a rectangle a fairly accurate retinotopic map would still develop as shown in Figure 1. However, if point stimuli were presented as inputs the retinotopic map would not emerge as demonstrated in Figure 2.

In order to demonstrate that the rough retinotopy imposed in the LISSOM architecture,is not sufficient for the map formation, the retinotopic map before training and the subsequent development for a few iterations are shown in Figure 3.

The LISSOM architecture requires afferent, lateral excitatory and lateral inhibitory connections for the retinotopic map to emerge. If only afferent connections are present, the projections become diffused and over time (iterations) becomes non-selective of the inputs. If only afferent and lateral excitatory connections are present, the outputs spread indiscriminately and the retinotopic map developed is meaningless. On the other hand, if only afferent and lateral inhibitory connections are present, due to the lack of reinforcement from the neighboring excitatory neurons in the output layer, the map developed again becomes diffused. The inhibitory lateral connections now uniformly inhibit almost all the neurons (due to the large inhibitory radius) and do not play a role in the map development. These simulation results are shown in Figure 4.

The extent of lateral connections defined by their respective radii also influences the map formation. On decreasing the lateral inhibitory radius, discontinuities are introduced in the developed map. The extent of these discontinuities depend of the extent of decrease in the inhibitory radius as shown in Figures 5, 6. These discontinuities occur because the map spread, permitted by the afferent connections, are not kept in check due to the relatively smaller inhibitory radius.

On the other hand, on increasing the lateral excitatory radius, the developed map spreads more than necessary and the neurons are selective to fewer meridional angles. The extent of this spread is dependent on the extent of increase in the excitatory radius, as shown in Figure 7, 8. This spread occurs because the large excitatory radius reinforces to a greater extent(spatially) the spread allowed by the afferent connections. An additional consequence is that the area occupied by the central, meridional angle non-sensitive region also increases.

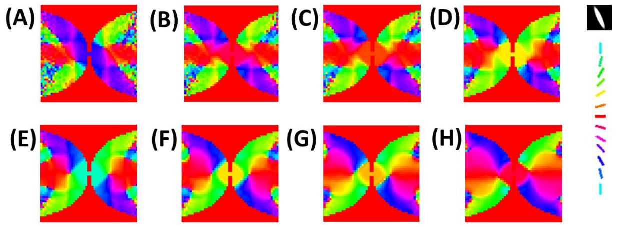

Figure 1. V1 map development when the inputs given to the LISSOM are ellipses of varying dilation and rotation: Meridional angle preference (color coded) at (A) 200, (B) 300, (C) 400, (D) 500, (E) 600, (F) 700, (G) 800, (H) 900 iterations respectively. This result demonstrates that it is the nature of the object in the inputs (i.e its rotations and dilations) that is necessary for the map formation rather than the shape of the object itself.

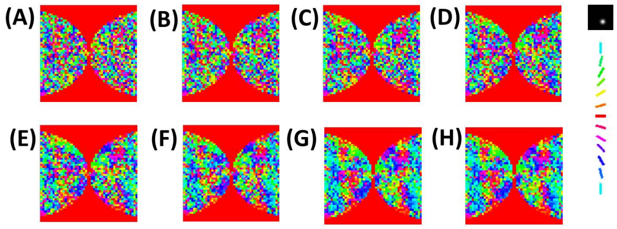

Figure 2. V1 map development when the inputs given to the LISSOM are point inputs occurring at random locations in the input space : Meridional angle preference (color coded) at (A) 200, (B) 300, (C) 400, (D) 500, (E) 600, (F) 700, (G) 800, (H) 900 iterations respectively. This result also demonstrates that it is the nature of the object in the inputs (i.e its rotations and dilations) that is necessary for the map formation rather than the shape of the object itself.

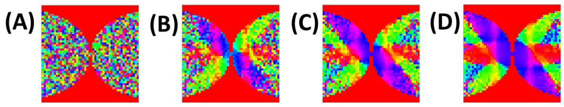

Figure 3. V1 map development : Meridional angle preference (color coded) at (A) 1, (B) 50, (C) 100, (D) 150 iterations respectively. This result demonstrates that the rough retinotopy imposed in the LISSOM architecture,is not sufficient for the map formation.

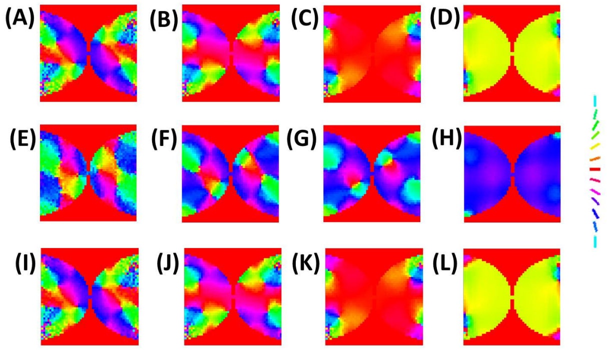

Figure 4. V1 map development : Meridional angle preference (color coded) at (A) 200, (B) 300, (C) 400, (D) 500 iterations respectively for excitatory and inhibitory strengths set to 0; at (E) 50, (F) 100, (G) 150, (H), 200 iterations respectively for inhibitory strength set to 0; at (I) 200, (J) 300, (K) 400, (L) 500 iterations respectively for excitatory strength set to 0. This result demonstrates that each of the 3 connection types: afferent, lateral excitatory, and later inhibitory are vital for the final map formation.

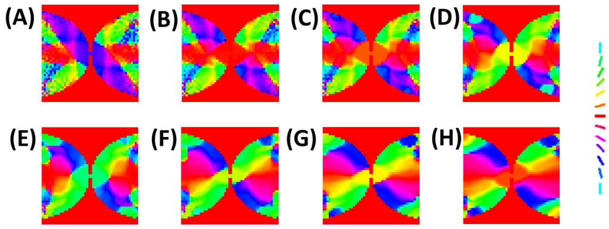

Figure 5. V1 map development with the maximum inhibitory radius (rad_I) set to 0.45 : Meridional angle preference (color coded) at (A) 200, (B) 300, (C) 400, (D) 500, (E) 600, (F) 700, (G) 800, (H) 900 iterations respectively. This result demonstrates that decreasing the maximum inhibitory radius (rad_I) results in discontinuities in the final map formed.

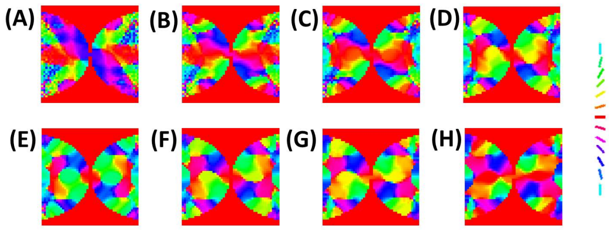

Figure 6. V1 map development with the maximum inhibitory radius (rad_I) set to 0.25 : Meridional angle preference (color coded) at (A) 200, (B) 300, (C) 400, (D) 500, (E) 600, (F) 700, (G) 800, (H) 900 iterations respectively. This result demonstrates that decreasing the maximum inhibitory radius (rad_I) results in discontinuities in the final map formed.

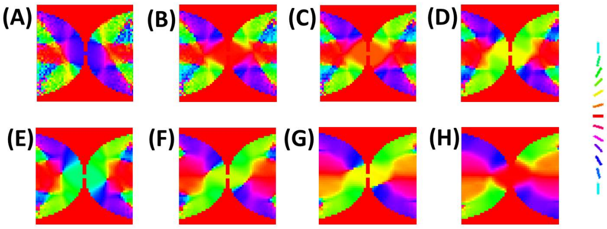

Figure 7. V1 map development with the maximum excitatory radius (rad_E) set to 0.1 : Meridional angle preference (color coded) at (A) 200, (B) 300, (C) 400, (D) 500, (E) 600, (F) 700, (G) 800, (H) 900 iterations respectively. This result demonstrates that increasing the maximum excitatory radius (rad_E) results in an additional (beyond requirement) spread in the final map formed.

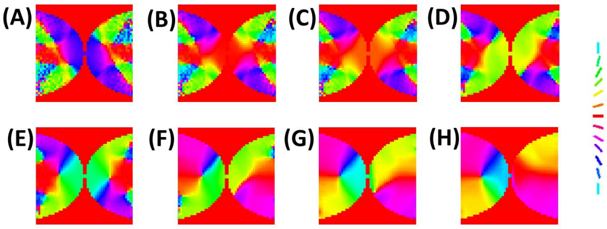

Figure 8. V1 map development with the maximum excitatory radius (rad_E) set to 0.2 : Meridional angle preference (color coded) at (A) 200, (B) 300, (C) 400, (D) 500, (E) 600, (F) 700, (G) 800, (H) 900 iterations respectively. This result demonstrates that increasing the maximum excitatory radius (rad_E) results in an additional (beyond requirement) spread in the final map formed.
